# Environmental conditions and herbivore biomass determine coral reef benthic community composition: implications for quantitative baselines

**DOI:** 10.1007/s00338-018-01737-w

**Published:** 2018-10-04

**Authors:** James P. W. Robinson, Ivor D. Williams, Lauren A. Yeager, Jana M. McPherson, Jeanette Clark, Thomas A. Oliver, Julia K. Baum

**Affiliations:** 1 Department of Biology, University of Victoria, PO BOX 1700, Station CSC, Victoria, BC V8W 2Y2 Canada; 2 Lancaster Environment Centre, Lancaster University, Lancaster, LA1 4YQ UK; 3 Ecosystem Science Division, Pacific Islands Fisheries Science Center, National Oceanic and Atmospheric Administration, 1845 Wasp Boulevard, Building 176, Honolulu, HI USA; 4 Department of Marine Science, University of Texas at Austin, Port Aransas, TX 78373 USA; 5 Center for Conservation Research, Calgary Zoological Society, 1300 Zoo Road NE, Calgary, AB T2E 7V6 Canada; 6 Department of Biological Sciences, Simon Fraser University, 888 University Drive, Burnaby, BC V5A 1S6 Canada; 7 Joint Institute for Marine and Atmospheric Research, University of Hawaìi at Mānoa, Honolulu, HI USA; 8 National Center for Ecological Analysis and Synthesis, University of California Santa Barbara, 735 State St #300, Santa Barbara, CA 93101 USA

**Keywords:** Macroecology, Biophysical, Grazing, Spatial scale, Top-down control, Decoupling, Abiotic forcing, Boosted regression trees

## Abstract

Our ability to understand natural constraints on coral reef benthic communities requires quantitative assessment of the relative strengths of abiotic and biotic processes across large spatial scales. Here, we combine underwater images, visual censuses and remote sensing data for 1566 sites across 34 islands spanning the central-western Pacific Ocean, to empirically assess the relative roles of abiotic and grazing processes in determining the prevalence of calcifying organisms and fleshy algae on coral reefs. We used regression trees to identify the major predictors of benthic composition and to test whether anthropogenic stress at inhabited islands decouples natural relationships. We show that sea surface temperature, wave energy, oceanic productivity and aragonite saturation strongly influence benthic community composition; overlooking these factors may bias expectations of calcified reef states. Maintenance of grazing biomass above a relatively low threshold (~ 10–20 kg ha^−1^) may also prevent transitions to algal-dominated states, providing a tangible management target for rebuilding overexploited herbivore populations. Biophysical relationships did not decouple at inhabited islands, indicating that abiotic influences remain important macroscale processes, even at chronically disturbed reefs. However, spatial autocorrelation among inhabited reefs was substantial and exceeded abiotic and grazing influences, suggesting that natural constraints on reef benthos were superseded by unmeasured anthropogenic impacts. Evidence of strong abiotic influences on reef benthic communities underscores their importance in specifying quantitative targets for coral reef management and restoration that are realistic within the context of local conditions.

**Electronic supplementary material:**

The online version of this article (10.1007/s00338-018-01737-w) contains supplementary material, which is available to authorized users.

## Introduction

Coral reef benthic communities are influenced by abiotic and top-down controls operating across a range of spatial scales (Mumby et al. 2006; Williams et al. 2013, 2015a). Interactions between fine-scale physical influences, such as wave exposure, and biotic influences, such as herbivorous grazing, are powerful structuring influences at local scales (Rasher et al. 2012), whereas large-scale abiotic influences may dominate at regional or global extents (Gove et al. 2013). Beyond the interplay between biotic and abiotic factors, anthropogenic disturbances can now also profoundly alter macroecological patterns, such that chronic anthropogenic stress can ‘decouple’ benthic organisms from their environment, rendering abiotic and biotic processes inaccurate predictors of benthic community structure (Williams et al. 2015a). As human impacts become more severe and widespread (Hughes et al. 2017), our understanding of altered benthic states will require empirical measures of the relative influences of abiotic and biotic processes across reef regions, set within the context of chronic stress.

In addition to the scleractinian corals that are the foundation of coral reef ecosystems, reef benthos is often also composed of crustose coralline algae (CCA), as well as turf and fleshy macroalgae. Hard coral and crustose coralline algae deposit calcium carbonate to form a structural reef architecture, whereas turf and fleshy macroalgae occupy coral settlement space and overgrow dead coral structures, although some macroalgal species also deposit carbonate (McCook et al. 2001). Examples from the Pacific Ocean show that the relative dominance of calcifying reef builders (hard coral, CCA) and non-calcifying algal organisms (turf and fleshy macroalgae) shifts along anthropogenic (Barott et al. 2012) and environmental gradients (Williams et al. 2015a), suggesting that reef benthos can exist in multiple regimes (Knowlton 1992) rather than only hard coral- or fleshy algal-dominated states (McManus and Polsenberg 2004). Thus far, however, the influences of abiotic factors and grazing at ‘macroscales’ (i.e., across biogeographic regions) have only been considered independently of one another and it is unclear whether there are potential interactions between them.

Grazing effects on coral reef benthic composition appear to vary widely across spatial scales. Small-scale experimental studies indicate that benthic community composition is strongly linked to the biomass (Mumby et al. 2006) and diversity (Burkepile and Hay 2008; Rasher et al. 2013) of herbivorous fishes, which maintain algal communities in cropped states that are likely to be relatively benign for coral growth and recruitment (Green and Bellwood 2009). The role of herbivorous fish biomass at macroecological scales is more uncertain, with correlative analyses providing examples of positive (Jouffray et al. 2015; Heenan and Williams 2013), negative (McCauley et al. 2014) and insignificant (Carassou et al. 2013; Suchley et al. 2016) influences of herbivore biomass on the promotion of calcifier cover or control of algal abundances. The disconnect between small-scale experiments and large-scale observations may be due to important but unquantified abiotic influences that, for example, place natural limits on recoverable levels of coral cover.

Indeed, there is evidence that diverse abiotic factors can influence coral reef benthic community composition. Natural variability in wave energy has recently been shown to influence local habitat suitability for coral survival (Gove et al. 2015) and grazers’ foraging ability (Bejarano et al. 2017), with exposed reefs generally characterized by low cover of calcifying organisms and a benthic community dominated by low-lying algal organisms (Williams et al. 2013, 2015a). Across regions and oceans, latitudinal gradients in the distribution of hard coral, CCA and algal cover likely reflect positive influences of sea surface temperature and the bottom-up influence of oceanic productivity on the growth rates of calcifying organisms, with coral and CCA cover declining from equatorial reefs to reefs in subtropical latitudes (Barott et al. 2012; Williams et al. 2015a). The availability of dissolved aragonite is strongly associated with calcification rates (Gattuso et al. 1998), and yet, despite evidence that aragonite saturation state can vary naturally among regions (Kuchinke et al. 2014), biochemical influences on reef benthic condition remain untested at large scales. Thus, reef benthic composition may be largely determined by local abiotic conditions, which derive from large-scale oceanographic processes. Yet, because all large-scale studies examining abiotic drivers to date have all focused solely on these drivers, it remains unclear how local grazing effects might modify, disrupt or enhance environmental constraints.

Additionally, temporal shifts from coral to algal dominance within a location may be associated with increased anthropogenic disturbances (e.g., sedimentation, pollution, overexploitation of grazers, habitat destruction, heat stress) that disrupt abiotic and top-down controls (Hughes et al. 2003; Graham et al. 2015). Although phase shifts from coral to algal states have been clearly documented on heavily degraded Caribbean reefs (Hughes et al. 2010) and following climate-driven thermal stress events in the Western Indian Ocean (Graham et al. 2015), in the Pacific, algal-dominated states can also occur on unimpacted remote reefs (Vroom and Braun 2010), and anthropogenic pressures may alter coral, CCA and fleshy algal abundances to produce multiple reef regimes (Jouffray et al. 2015). At local scales, site-level shifts in benthic state have been linked to fishing pressure and water quality metrics in some locations (e.g., Jouffray et al. 2015), but at larger scales—e.g., islands—human impacts have been measured by comparing benthic states between uninhabited and inhabited reefs (Williams et al. 2015a). In this way, reef benthic communities have been shown to ‘decouple’ from natural abiotic processes on inhabited Pacific islands, likely due to reorganization of dominant benthic taxa (Williams et al. 2015a). Nevertheless, it remains unclear whether abiotic decoupling is detectable at smaller scales (i.e., site level), or whether intra-island differences in benthic state are partly attributable to gradients in herbivore exploitation.

Macroecological tests of competing abiotic, biotic and anthropogenic influences can help to resolve how reef conditions determine benthic composition. Such analyses enable meaningful comparisons of reef regions and thus improve our understanding of anthropogenic impacts. Here, we combine site-level underwater image and visual census data with remotely sensed environmental data to test the relative influence of abiotic and biotic processes on the relative abundances of calcifying hard coral and CCA versus non-calcifying turf and macroalgal organisms at 34 Pacific islands and atolls. Surveyed islands display substantial spatial heterogeneity in abiotic conditions, ranging from warm equatorial reefs to cool subtropical reefs (Williams et al. 2015a), from oligotrophic island chains to atolls in productive upwelling zones, and including substantial intra-island and inter-island variability in wave energy (Gove et al. 2013). The islands also form several distinct island groups, each of which has large gradients in fishing pressure and in herbivore biomass (Heenan et al. 2016). We quantified the relative importance of 4 abiotic variables (temperature, oceanic productivity, wave energy and aragonite saturation state) and 3 grazing variables (grazer, scraper/excavator and browser herbivore biomass) in predicting fine-scale patterns in the relative abundance of calcifying (hard coral and CCA) and algal (turf and macro) organisms—the reef-builder index—over the extent of the Central Pacific Ocean (~ 43° latitude × 61° longitude). While recognizing that coral reef benthos comprises of numerous species and taxonomic groups, we have used a univariate indicator (building on Smith et al. 2016) because it provides both a clear delineation between two major categories of reef benthos and a tractable means of assessing major types of drivers (abiotic, biotic and anthropogenic) across reef regions. We hypothesized that coral reef benthic community composition would be primarily predicted by abiotic factors, because these set fundamental constraints on the growth rates of competing benthic organisms, with secondary influences from grazers in promoting calcified states. By fitting statistical models separately to uninhabited and inhabited islands, we also considered how predicted relationships might decouple under a chronic disturbance regime.

## Methods

### Coral reef data and treatment

**Fig. 1:**
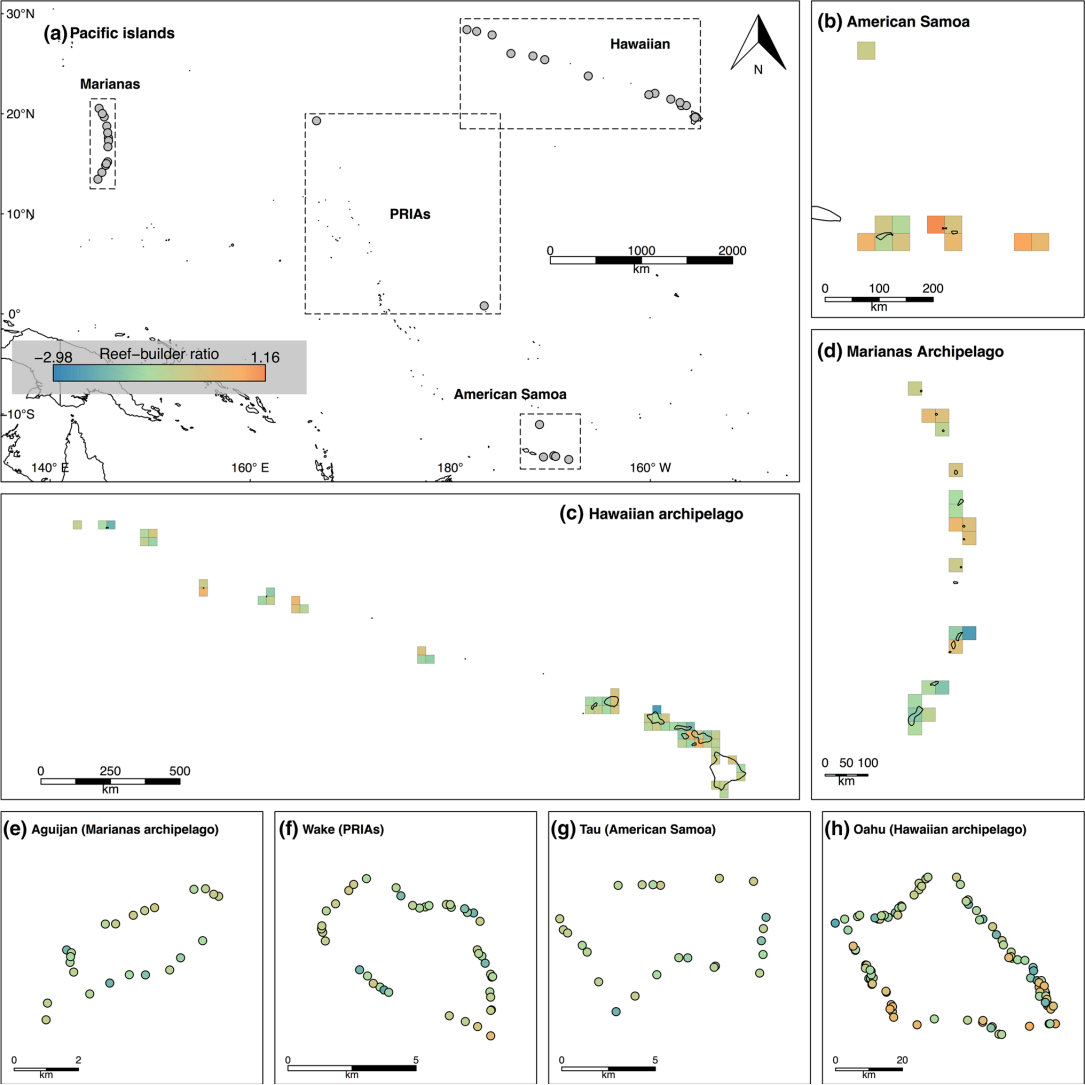
Spatial variation in reef benthic community composition across 34 Pacific Islands and atolls (**a**). Each cell is colored by the reef-builder index value (red = calcifier-dominated; blue = algal-dominated) averaged across all sites within 1024 km^2^ grid cells, for American Samoa (*n* = 5) (**b**), Marianas archipelago (*n* = 13) (**c**) and Hawaiian archipelago (*n* = 14) (**d**), and for site-level variation across one representative island from each island group: Aguijan (**e**), Wake (**f**), Tau (**g**) and Oahu (n), with points representing UVC sites colored by ratio values

Data on benthic cover and herbivorous fish assemblages were collected between 2010 and 2014 by trained scientific divers of the Coral Reef Ecosystem Program (CREP) of NOAA’s Pacific Island Fisheries Science Center. Underwater visual censuses (UVC) and benthic photoquadrats (PQs) were carried out at 34 US-affiliated tropical Pacific islands and atolls, encompassing the Hawaiian and Marianas archipelagoes, American Samoa and the Pacific Remote Island Areas (PRIAs); this region spans gradients of human population density, sea surface temperature and oceanic productivity (Fig. 1; Supplementary Material 2, Table A1) (Coral Reef Ecosystem Program). UVC observations were used to estimate herbivorous fish biomass, and PQs provided estimates of mean percent cover of broad taxonomic groups (Supplementary Material 3).

We characterized variation among benthic communities using three metrics: calcifying organism cover (hard corals, CCA), fleshy algal organism cover (turf and non-calcareous macroalgae) and, as an integrated measure of reef benthic state, the ratio of calcifier to fleshy algal cover. After transforming the ratio onto a log_10_ scale (hereafter the reef-builder index), positive values indicate sites dominated by calcifying organisms (> 50% calcifier cover) and negative values indicate sites dominated by algal organisms (> 50% algal cover) (Supplementary Material 1, Figs. A1, A2). Benthic substrate composed of sand and sediment was omitted from these calculations, and thus, the reef-builder index represents the relative cover of major benthic taxa rather than absolute abundances. The index also combines benthic taxa that have distinct ecological functions. For calcifiers, positive values may represent high cover of coral or CCA and thus represents calcification potential rather than directly correlating to calcification rates (Smith et al. 2016). For algae, negative values may represent high cover of turf algae or macroalgae and thus do not distinguish between reefs with cropped turf habitats and those dominated by macroalgae (Supplementary Material 6). We tested the sensitivity of the reef-builder index to macroalgal-dominated sites (i.e., as opposed to those with a mix of turf and macroalgae) by recomputing estimates after excluding macroalgal cover.

### Predictor variables

Our biotic grazing predictor, herbivorous fish grazing pressure, was represented by site-level biomass estimates calculated from the UVC fish observations. Biomass is linked to energy expenditure and, as bite and foraging rates scale allometrically with body size, grazing biomass is widely used as a proxy for grazing pressure (Nash et al. 2013, 2015). Furthermore, extensive observations of herbivorous fish feeding mechanisms and behaviors have been used to classify these species into a number of broad functional groups representing distinct grazing functions. Adapted from Green and Bellwood (2009) and Yeager et al. (2017a), we classified herbivorous fish species as: (1) croppers, which feed primarily on turf algal assemblages, including detritus, with minimal impacts to the coral substrate; (2) scrapers and excavators, which consume algae, detritus and coral by scraping or removing the upper layer of the reef substrate; (3) browsers that primarily feed on fleshy macroalgae and do not impact coral substrate (Supplementary Material 3, Table A2). For each functional group, herbivore biomass was used as a proximate measure of the strength of herbivory.

To examine abiotic influences on benthic community structure, we compiled remote sensing data for sea surface temperature, net primary productivity, wave energy and aragonite saturation, because these four covariates have previously been shown to influence benthic community composition. We obtained average weekly minimum SST (°C) estimates from the National Oceanographic Data Center’s Coral Reef Temperature Anomaly Database (CoRTAD), based on AVHRR Pathfinder data between 1982 and 2008 at a ~ 4.6 × 4.6 km resolution (http://www.nodc.noaa.gov/SatelliteData/Cortad). Net primary productivity (mg C m^−2^ d^−1^) estimates were extracted from NOAA CoastWatch based on satellite measurements of photosynthetically available radiation (NASA’s SeaWiFS), SST (NOAA’s National Climatic Data Center Reynolds Optimally Interpolated SST) and chlorophyll a concentration (NASA Aqua MODIS) and were estimated every 8 d between 2002 and 2013 at a ~ 4.6 × 4.6 km resolution (http://coastwatch.pfeg.noaa.gov/erddap/griddap/erdPPbfp28day.graph) (Behrenfield and Falkowski 1997; Yeager et al. 2017b). UVC site estimates were the average across the time series (Supplementary Material 3). Although defined here as abiotic, our oceanic productivity metric is a proxy for phytoplankton availability and thus represents a bottom-up process. To determine wave energy, we extracted wave power hourly estimates from the global Wave Watch III model (Tolman 2014) at a 50 × 50 km resolution, forced with hindcast winds from 1979 to 2010 (Durrant et al. 2013). Aragonite saturation data were extracted at the site level from the 1° × 1° resolution GLODAPv2 ocean biochemistry climatology dataset (Lauvset et al. 2016) (Supplementary Material 3). These aragonite saturation state estimates (Ω_a_) were mapped to a global extent by data interpolation of CO_2_ chemistry samples collected from 724 large-scale oceanographic cruises between 1972 and 2013 (Olsen et al. 2016). UVC depth was also included as a predictor covariate to account for changes in water turbidity, light irradiance and water flow along the shallow depth gradient (0–30 m) (Williams et al. 2013). We initially considered island type (atoll, low island and high island), to account for variation in topography and terrestrial inputs, but found that it was a weak predictor (< 1.5% variable influence) and thus excluded this variable from our predictive models.

Finally, to assess potential decoupling of both abiotic and grazing influences, we classified islands into low (uninhabited islands and far from population centers) and high disturbance groups (inhabited islands and near to population centers) using criteria developed for previous analyses of the CREP dataset (Williams et al. 2015b) (Supplementary Material 2, Table S1). Human impacts were assigned at the island level and thus did not account for intra-island disturbance gradients across sites.

### Analyses

We used boosted regression trees (BRTs) to examine the relative strength of each covariate and all pairwise interactions in predicting the reef-builder index, calcifier cover and fleshy algal cover at fine scales (i.e., each site, ~ 353 m^2^) over an ocean basin extent. BRT models are regression tree ensembles constructed by building ‘trees’ sequentially where, at each stage, the next tree attempts to minimize the deviance of the residuals of the previous tree (Elith et al. 2008). Thus, boosting improves model predictive performance and robustness of single trees. BRTs provide a flexible method of modeling relationships between variables that can incorporate complex interaction effects, while also modeling nonlinear relationships (Elith et al. 2008), which have been detected in previous macroecological analyses of spatial variation in reef benthic cover (Jouffray et al. 2015; Heenan and Williams 2013). BRT performance was optimized by adjusting three model parameters: tree complexity (tc), which sets the number of nodes in each tree; learning rate (lr), which sets the importance of each tree added and so influences the number of trees included in each model; and bag fraction, which sets the proportion of the data utilized in each tree. We fitted models to all combinations of parameter values across tc (1–2–3–4–5), lr (0.01, 0.001, 0.0001) and bag fraction (0.25, 0.5, 0.75, 0.9) and selected the parameter set with the lowest mean predictive deviance as our final fitted model (Richards et al. 2012) (Supplementary Material 4, Table A3). BRTs were fitted to a normal distribution for the reef-builder index, and a Poisson distribution for percent cover estimates of calcifiers and fleshy algal taxa.

For each benthic response variable, BRTs were first fitted using the full dataset and then, to evaluate potential human-induced decoupling, separately to inhabited and uninhabited datasets (sensu Williams et al. 2015a). For all fitted models, we assessed the relative strengths of abiotic and grazing predictors by extracting the *gbm* measure of relative importance, which is scaled between 0% (weak influence) and 100% (strong influence). Additionally, relationships between the reef-builder index and predictors were visualized using partial dependency plots that show the fitted function while holding the effect of other predictors at their mean (Elith et al. 2008). Uncertainty in relative importance estimates and model predictions was quantified using bootstrapped 95% confidence intervals (Leathwick et al. 2006). Relative model performance was assessed by estimating the overall deviance explained and mean predictive deviance for each optimal model. Interactions between predictors were estimated using the *gbm*.*interactions* function in the *dismo* package (Hijmans et al. 2017), and we accounted for spatial autocorrelation using autocovariates to capture correlations in values between neighboring sites (Crase et al. 2012) (Supplementary Material 3).

All analyses were performed using R version 3.4.1 (R Development Core Team 2017), BRTs were fitted with the *gbm* (Ridgeway 2017) and *dismo* (Hijmans et al. 2017) packages, and we provide our data and code at an open-source repository (https://github.com/baumlab/Robinson-etal-2018-CoralReefs).

## Results

At 1566 sites across 34 islands and atolls spanning ~ 43° latitude by ~ 61° longitude, reef benthic states ranged from calcifier-dominated (219 sites (13.98%) at 20 islands; i.e., > 50% calcifying cover; 0.03 < reef-builder index < 2.06) to algal-dominated (1129 sites (72.09%) at 32 islands; i.e., > 50% fleshy algal cover; − 3 < reef-builder index < − 0.01) (Supplementary Material 2, Table A1). The remaining 13.9% of sites were dominated by neither hard coral nor fleshy algae, but rather by calciferous *Halimeda* algae, soft corals, sediment and unclassified material. Calcifiers typically occupied much less space than fleshy algae (median cover: calcified = 16.7%; algal = 67.3%), such that six islands in Hawaii (43% of all islands in this region) and seven islands in the Marianas (54% of all islands in this region) lacked any calcifier-dominated sites. In contrast, algal-dominated sites occurred on every island, rendering this the more common state across the Pacific (Fig. 1). Among algal-dominated sites, macroalgal-dominated reefs were rare (1.3% of sites with > 50% macroalgal cover) and, as such, negative reef-builder index values were largely representative of high turf cover reefs (Fig. A6).

**Fig. 2:**
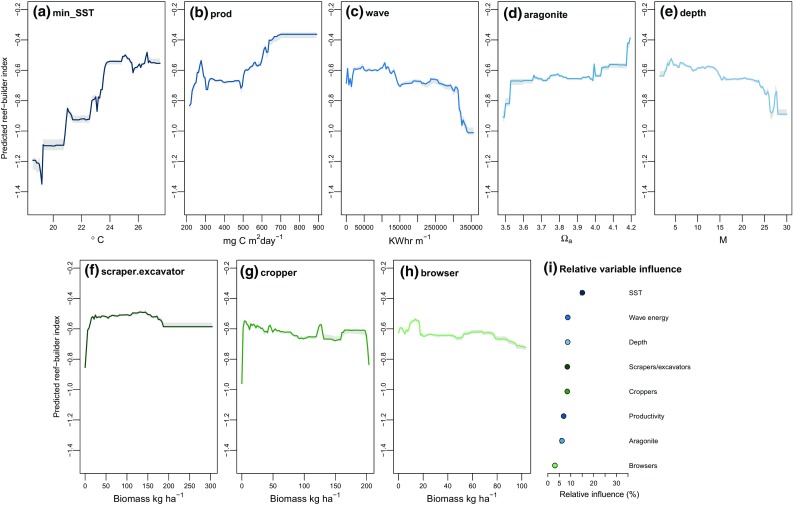
Partial dependence plots and relative importance values for each covariate. Partial dependence plots show predicted change in reef-builder index values along the range of each abiotic covariate (**a**–**e**) and biotic grazing covariate (**f**–**h**), with relative importance values (**i**). Fitted lines are predicted reef-builder index values across the range of each selected covariate, holding all other covariates to their mean and with data deciles indicating the distribution of original observations. Red dashed lines are smoothed LOESS functions, and shaded areas are 95% uncertainty envelopes generated from bootstrapped model predictions

Abiotic covariates were strong predictors of benthic community composition. Regions of high SST, oceanic productivity and aragonite saturation state, and low wave energy, were associated with higher reef-builder values (i.e., greater calcified cover and lower fleshy algal cover) (Fig. 2a–c). Along latitudinal temperature and productivity gradients, the occurrence of algal-dominated reefs was predicted at the lowest temperatures (< 21 °C) and productivities (< 300 mg C m^2^ d^−1^) (Fig. 2a, b). When modeled as the response, calcifier cover remained relatively invariant across temperatures with a mean predicted cover of 20%, whereas algal cover declined from 68 to 48% as temperature increased from 18.5 to 27.5 °C. Thus, high calcified cover at warmer reefs was due to declines in algal cover, which increased the relative abundance of coral and CCA. The model predicted higher cover of calcifying taxa at higher level of ocean productivity, increasing from 20 to 33% mean predicted cover over 500–700 mg C m^2^ d^−1^ (Supplementary Material 1, Fig. A4a, b). Wave energy also had a moderate influence on the reef-builder index, which decreased as wave energy increased (Fig. 2c), such that calcified cover was maximized at low wave energy sites (< ~ 25,000 KW h m^−1^) and algal cover highest at high wave energy sites (> 250,000 KW h m^−1^) (Supplementary Material 1, Figs. A4c, A5c). Aragonite saturation state had the weakest abiotic influence on the reef-builder index, but was a strong predictor of both calcifier cover and fleshy algal cover when these were modeled separately. Models predicted a gradual increase in calcified dominance with aragonite saturation state (Fig. 2d), with calcifier cover maximized at 45% on reefs with high (4.2 Ω_a_) aragonite saturation states and predicted fleshy algal cover reaching a peak of 65% at low (3.5 Ω_a_) saturation states (Supplementary Material 1, Figs. A4d, A5d).

The importance of grazing biomass in predicting the reef-builder index was generally lower than abiotic covariates, with scraper and excavator species and cropper species estimated as the fourth and fifth most important predictors (Fig. 2i). However, the predicted grazing effect was similar for both functional groups, whereby shifts to algal-dominated values (negative effect on reef-builder index) were only observed at low biomass estimates (< 20 kg ha^−1^), and herbivore biomass above this threshold had no further effect (Fig. 2f,g). Below 20 kg ha^−1^, cover values decreased (for calcifiers) or increased (for algae) by ~ 5% (Supplementary Material 1, Figs. A4f,g,h, A5f,g). Browsers had the weakest effect, with calcifier cover remaining steady across the browser biomass gradient (Fig. 2h; Supplementary Material 1, Fig. A4i).

**Fig. 3:**
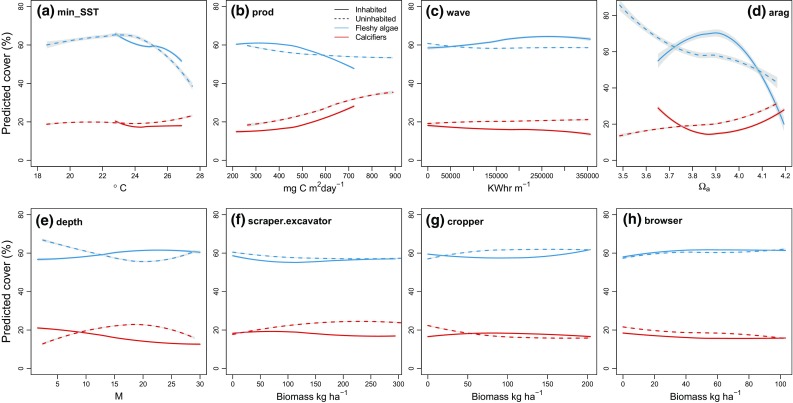
Effect of human disturbance on predicted covariate relationships. Partial dependence plots show predicted change in reef-builder index (red) and fleshy algal percent cover (blue) along the range of each abiotic covariate (**a**–**e**) and top-down biotic grazing covariate (**f**–**h**), for inhabited (solid line) and uninhabited (dashed line) reefs. Fitted lines are LOESS smoothed predicted values across the range of each selected covariate, holding all other covariates to their mean. Shaded areas represent ± 2 standard errors on LOESS fits

Abiotic and grazing relationships did not decouple at disturbed locations. BRTs fitted separately to inhabited and uninhabited island datasets identified similar functional relationships for almost every abiotic and biotic covariate (Fig. 3). Predicted relationships did, however, decouple along a depth gradient where, compared to uninhabited islands, inhabited reefs had greater calcifier cover at shallow depths (< 10 m) and lower calcifier cover below 15 m (Fig. 3e). Despite no clear decoupling of biophysical drivers, autocovariate relative importance values were highest in inhabited dataset BRTs and particularly strong in the inhabited reef-builder index (autocovariate relative importance = 43.1%) and calcifier cover (31.8%) models (Supplementary Material 4, Table S3), indicating that inhabited reef sites were more spatially autocorrelated than uninhabited reefs.

Deviance explained was ~ 45–58% for all models, indicating that BRTs performed equally in predicting fine-scale patterns in the reef-builder index, calcified cover and fleshy algal cover across different disturbance regimes (Supplementary Material 4, Table S3). Unexplained deviance was partly attributable to inaccurate predictions of the highest and lowest reef-builder index values, although there were no residual patterns in any benthic cover model (Supplementary Material 1, Fig. A3). Autocovariates were important predictors (relative importance: 3.9–43.1%) and were effective in reducing spatial autocorrelation in all BRTs (Moran’s *I* coefficient: − 0.03 to 0.08) (Supplementary Material 4, Table S3). Interactions were strongest between abiotic covariates and site depth, with higher SST, oceanic productivity and aragonite state values at shallower depths, while grazing covariates interacted weakly with each other (all pairwise interactions < 0.37) (Supplementary Material 5, Table S4).

## Discussion

We combined an expansive ecological monitoring dataset with remotely sensed environmental and anthropogenic covariates to show that on reefs with more than a minimal threshold of herbivore biomass, coral benthic community composition [measured as the ‘reef-builder index’, a composite indicator of the relative abundances of calcifiers (hard coral, CCA) and algae (turf and fleshy macroalgae)] was primarily predicted by natural variation in temperature, productivity, wave energy and aragonite saturation state. Calcifier-dominated reefs occurred in warm, productive regions on reefs with low wave energy and high aragonite concentrations. Herbivorous fishes were important influences on the reef benthos, with a loss of grazing pressure at low cropper and scraper and excavator biomass levels corresponding with a transition toward algal-dominated reefs. Our analyses suggest that abiotic conditions outweigh or match grazing pressure as predictors of the relative abundances of calcifying and algal organisms at the site-level scale and provide evidence of the nonlinear influence of grazing pressure on reef benthic community composition.

We found that abiotic factors were important predictors of benthic community state, with the influence of sea surface temperature stronger than all other covariates. Shifts from negative (algal-dominated) to positive (calcifier-dominated) reef-builder values tracked increases in sea surface temperature, as in Williams et al. (2015a), representing a latitudinal gradient in the relative abundance of reef calcifiers that is likely linked to energetic constraints on the growth rates of calcifying organisms (Johannes et al. 1983). The mechanisms by which other abiotic processes influence coral reef ecosystems are less clear. High chlorophyll a concentrations, which are indicative of enhanced near-shore phytoplankton biomass (Gove et al. 2016), have been positively associated with biomass of sharks, planktivorous and piscivorous teleost fishes (Nadon et al. 2012; Williams et al. 2015b), suggesting that increases in particulate food availability (Leichter et al. 1998) and/or background nutrient supply (Burkepile et al. 2013) can indirectly promote coral and CCA cover on Pacific reefs (Williams et al. 2015a). Calcifier cover also increased with aragonite saturation state, which demonstrates an empirical link between reef benthic structure and carbonate availability at an oceanic scale. Aragonite estimates were, however, time-averaged and thus may mask fine-scale spatial variation in ocean acidification rates (Hoegh-Guldberg et al. 2007), which limited our ability to detect fine-scale shifts in calcification ability.

Wave energy was moderately important in predicting reef-builder values, consistent with evidence that habitat suitability is a key influence on benthic community composition between sites (Williams et al. 2013) and among islands (Williams et al. 2015a). Several mechanisms may link wave action to benthic composition. Coral organisms, particularly branching growth forms, are vulnerable to dislodgement, breakage and scour in high-energy environments (Madin and Connolly 2006), which might additionally inhibit grazing activity (Bejarano et al. 2017). Moderate wave exposure may also raise turf productivity (Crossman et al. 2001), resulting in algal-dominated reefs that can support large grazer populations (Heenan et al. 2016). Despite limitations in the spatial resolution of remotely sensed covariates (Supplementary Material 6), the combined influence of abiotic processes was consistently stronger than top-down biotic covariates, in agreement with recent studies that have highlighted the significant roles played by biophysical factors in structuring coral reef benthic communities (Madin and Connolly 2006; Williams et al. 2015a).

We also found that grazing pressure by cropper, scraper and excavator species outweighed that of browsing herbivores, as well as influences of productivity and wave energy. Such high relative importance suggests that croppers, scrapers and excavators play an important role in promoting coral recruitment and controlling algal cover across biogeographic regions and that they might play a larger role than browser functional groups. Large-bodied fishes, such as scrapers and excavators, are often preferentially targeted by fishers (Robinson et al. 2017), suggesting that exploitation likely underpins the observed gradient in grazing pressure. These results align with experimental site-level grazer exclusion studies indicating that scraper biomass limits macroalgal cover (Mumby et al. 2006) and that the presence of both cropper and scraper species promotes coral cover (Burkepile and Hay 2008), as well as observational evidence of positive associations between site-level estimates of coral cover and scraper biomass (Heenan and Williams 2013; Jouffray et al. 2015; Williams et al. 2016). Weak influences of browsing herbivores, which feed on macroalgae, likely reflect the low incidence of macroalgal-dominated reefs in our dataset, though future studies that are able to assess influences on finer-scale benthic groups and, for example, distinguish between turf and macroalgae, may be able to shed further insight. By relying on biomass as a proxy for grazing pressure, our analysis was unable to account for natural variation in grazing intensity due to environmental differences, such as lower grazing rates in cooler regions (Bruno et al. 2015), or for behavioral differences within functional groups (Streit et al. 2015). Indeed, herbivore biomass itself has been shown to track temperature gradients (Heenan et al. 2016), meaning that grazing might become decoupled from algal abundances, particularly if algal dominance shifts from turf to macroalgae (Supplementary Material 6). Further investigation into natural variation in grazing intensity across regions with different environmental regimes will help to connect experimental grazing studies with correlational patterns such as ours. However, as several abiotic covariates were consistently stronger predictors of the reef-builder index than grazing biomass, we suggest that, at the scale of our study, benthic composition of a given reef is primarily determined by environmental conditions rather than grazing capacity, given a minimum threshold of grazer presence.

Despite previous evidence that biophysical benthic drivers decouple across Pacific islands (Williams et al. 2015a), our analyses showed that biophysical and grazing relationships were similar at inhabited and uninhabited reefs. The discrepancy between Williams et al. (2015a) and our analysis is likely largely a problem of scale. Intra-island gradients in biophysical drivers (Gove et al. 2015) and human stressors (including herbivore exploitation) drive site-level heterogeneity in benthic community compositions that may be obscured in island-scale analyses (Williams et al. 2015a). Thus, at finer scales, abiotic influences remain important predictors of disturbed reef systems, perhaps in part because inhabited islands are larger than uninhabited atolls and thus tend to be characterized by substantially more benthic and biophysical variability. We were also able to account for exploitation gradients that alter grazing control and that might therefore have been part of the reason for decoupling. Nevertheless, strong spatial autocorrelation at inhabited islands suggests that anthropogenic stressors do drive benthic degradation at a sub-island scale (i.e., on nearby reefs) and can weaken the influence of abiotic and grazing processes. Thus, relationships were not decoupled, but instead weakened as unmeasured anthropogenic stressors homogenized benthic communities among neighboring reefs on inhabited islands. Combining herbivore biomass with fine-scale indices of terrestrial pollution to predict benthic states within islands (e.g., Jouffray et al. 2015) in future studies would facilitate understanding of which scales are most relevant for human impacts. Indeed, improving the temporal resolution of herbivore surveys and spatial and temporal grain of remotely sensed abiotic covariates will greatly advance our understanding of scale dependence in benthic drivers (Supplementary Material 6). Such approaches are particularly critical in the context of ongoing warming and acidification of reef environments, which further confound empirical assessments of anthropogenic influences as local impacts become superseded by global stressors (Bruno and Valdivia 2016).

Understanding the relative influences of abiotic and biotic factors on benthic community structure, and potential decoupling of those relationships, can provide insights into which components of reef resilience might be most effectively managed. Our results suggest that biophysical context is likely key in controlling the relative abundance of calcifiers and algal organisms and thus is a primary determinant of reef state at macroecological scales. For example, some remote Hawaiian coral reefs which are algal-dominated irrespective of grazer biomass have challenged perceptions that healthy reefs are always coral-dominated (Vroom and Braun 2010; Helyer and Samhouri 2017), while others have demonstrated that macroalgal taxa have broad functional roles, ranging from fleshy algal food for browsing herbivores (Streit et al. 2015) to reef sediment production by calcareous *Halimeda* (Perry et al. 2015). As such, fine-scale analysis of variation in algal community structure, including transitions from turf to macroalgal regimes (Jouffray et al. 2015), will advance our understanding of the health and functioning of algal reefs. Alternatively, in warm and productive regions, healthy and diverse grazing communities confer resilience after loss of coral cover following disturbance events (Cheal et al. 2010; Graham et al. 2015). Such distinctions can be used to inform quantitative baseline states for degraded reef systems across environmental gradients, which will be vastly improved by integration of local abiotic constraints with grazing capacity.

Irrespective of environmental conditions, nonlinearities in benthic community composition–grazing relationships show that areas of extremely low herbivore biomass are characterized by algal-dominated states, consistent with the evidence of thresholds in reef benthic state at low grazer biomass (Graham et al. 2015; Jouffray et al. 2015). Such grazing tipping points, which have previously only been demonstrated at small scales (Rasher et al. 2013; Holbrook et al. 2016) or within regions (Jouffray et al. 2015), provide tangible targets for conserving grazing function on exploited reefs. Consideration of grazing thresholds may help to resolve uncertainty around the effectiveness of management strategies that aim to protect reef benthos by promoting herbivorous grazing in marine protected areas. For example, examples of high grazing rates enhancing coral growth and recruitment (Mumby et al. 2007; Rasher et al. 2012) appear at odds with studies reporting no effect of protection status on benthic state (Jones et al. 2004), while perceived management ineffectiveness in promoting coral recovery may arise when herbivore populations remain at low levels (Huntington et al. 2011; Carassou et al. 2013). Our results suggest that, when environmental conditions promote cover of calcifying organisms, restoring grazing function of cropper, scraper and excavator species at heavily exploited reef sites that support very low herbivore populations and, in less degraded regions, preventing depletion of grazer populations below a threshold ~ 10–20 kg ha^−1^ could be effective in controlling potentially problematic algae and maintaining dominance of reef builders.

In coral reef ecosystems, the roles of abiotic, biotic and anthropogenic processes in driving macroecological patterns have usually been considered independently. For example, variation in Pacific reef benthic cover has been examined among regions in the context of human presence alone (Smith et al. 2016) or of humans and biophysical forces (Williams et al. 2015a) and, within regions, in the context of either grazing biomass (Jouffray et al. 2015) or abiotic drivers (Sandin et al. 2008) along human disturbance gradients, but rarely for all three components or across different regions. Here, we show how large-scale abiotic gradients set constraints on coral reef benthic community composition and are modified by local biophysical processes and herbivore grazing pressure. Our results provide a foundation for a unified understanding of the strength of abiotic and biotic controls on reef benthic communities and predict abiotic relationships that can help inform expectations of both contemporary baselines and future benthic states for Pacific reefs, as species respond to anthropogenic warming and ocean acidification (Hughes et al. 2017). Understanding constraints on benthic community configurations will be further advanced by combining fine-scale remote sensing data (e.g., Wedding et al. 2018) with replicated ecological observations and testing for scale dependence in potential decoupling of benthic drivers. Such examination of spatial and temporal variation in the biophysical and grazing factors that structure reef benthos across scales will help to ensure that anthropogenic impacts are framed in the correct abiotic context.

## Electronic supplementary material

Below is the link to the electronic supplementary material.

Supplementary material 1 (PDF 952 kb)

Supplementary material 2 (XLSX 40 kb)

Supplementary material 3 (DOCX 119 kb)

Supplementary material 4 (XLSX 38 kb)

Supplementary material 5 (XLSX 31 kb)

Supplementary material 6 (DOCX 107 kb)
