# Supplementary material for: Environmental conditions and herbivore biomass determine coral reef benthic community composition: implications for quantitative baselines
Source: Coral Reefs. 2018 Oct 4;37(4):1157–68. doi: 10.1007/s00338-018-01737-w (PMC6404665; doi:10.1007/s00338-018-01737-w)
Supplement: Supplementary file 1 — Supplementary material 1 (PDF 952 kb) [file 338_2018_1737_MOESM1_ESM.pdf]

Appendix 1: Supplementary figures

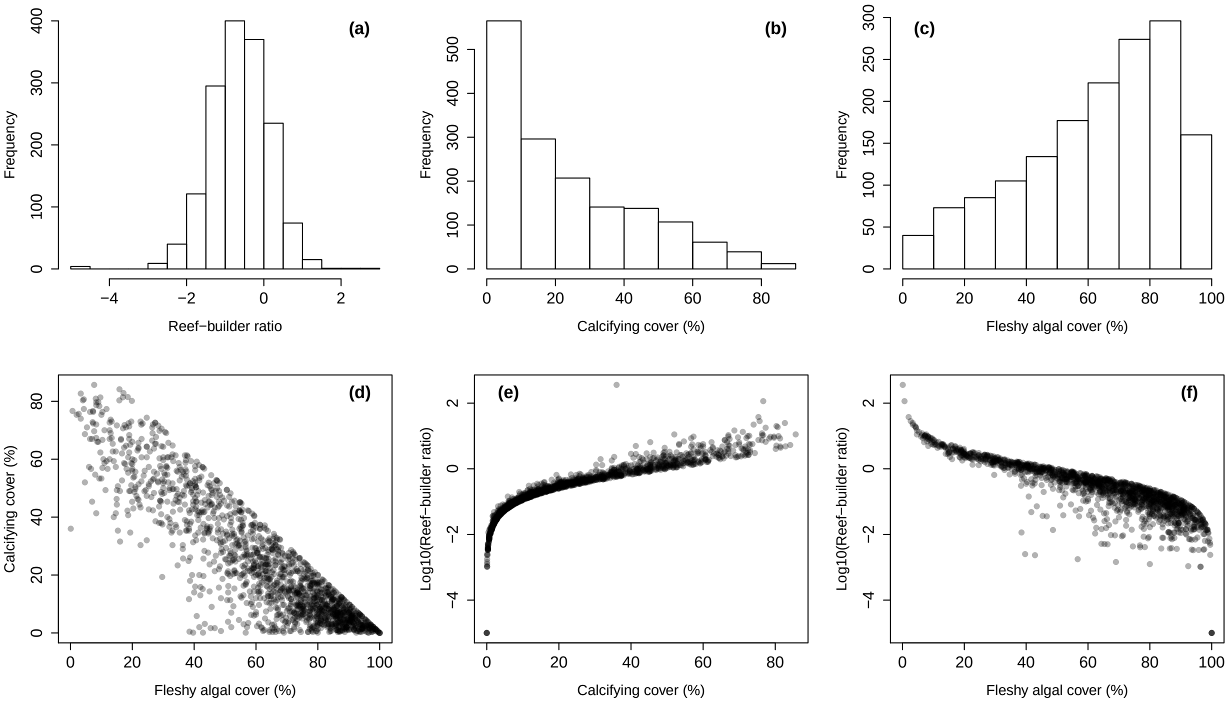

Figure A1. Histogram of reef-builder index values (a), calcifying cover (b) and fleshy algal cover (c), and scatterplots of calcifying vs. fleshy algal cover (%) (d), reef-builder index vs. calcifying cover (%) (e), and reef-builder index vs. fleshy algal cover (%) (f).

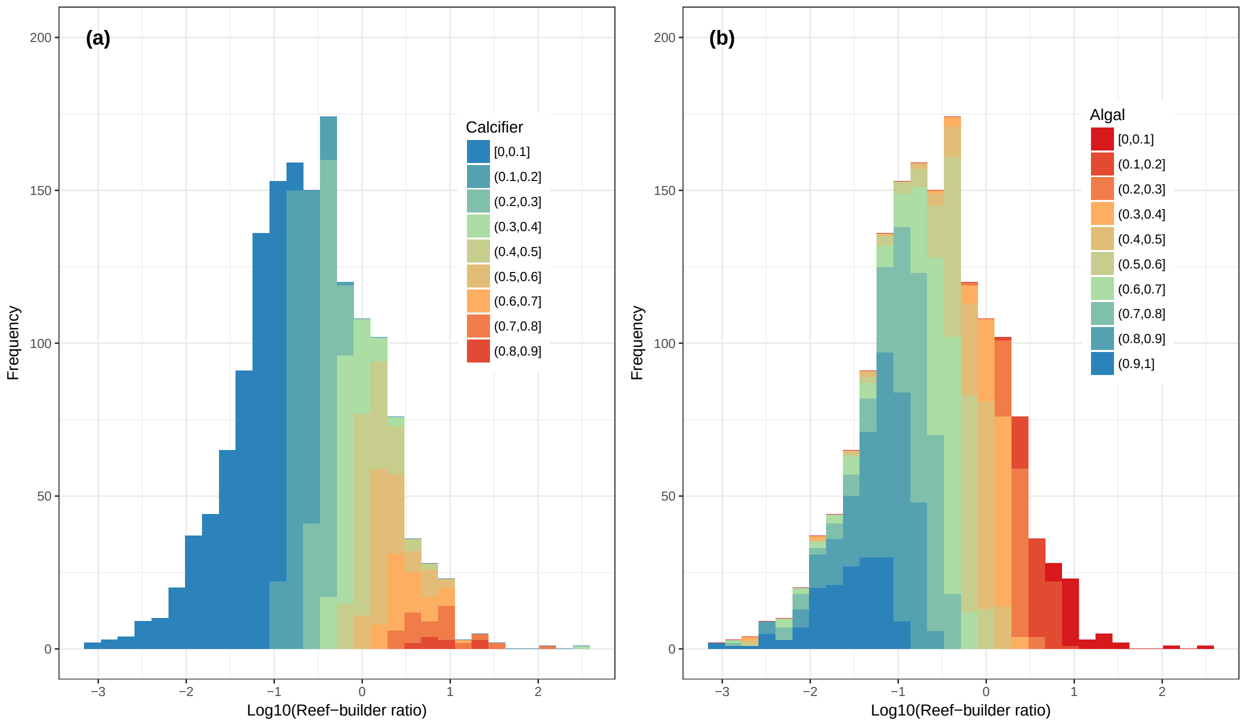

Figure A2. Histogram of reef-builder index values filled by corresponding calcified cover (a) and fleshy algal cover (b).

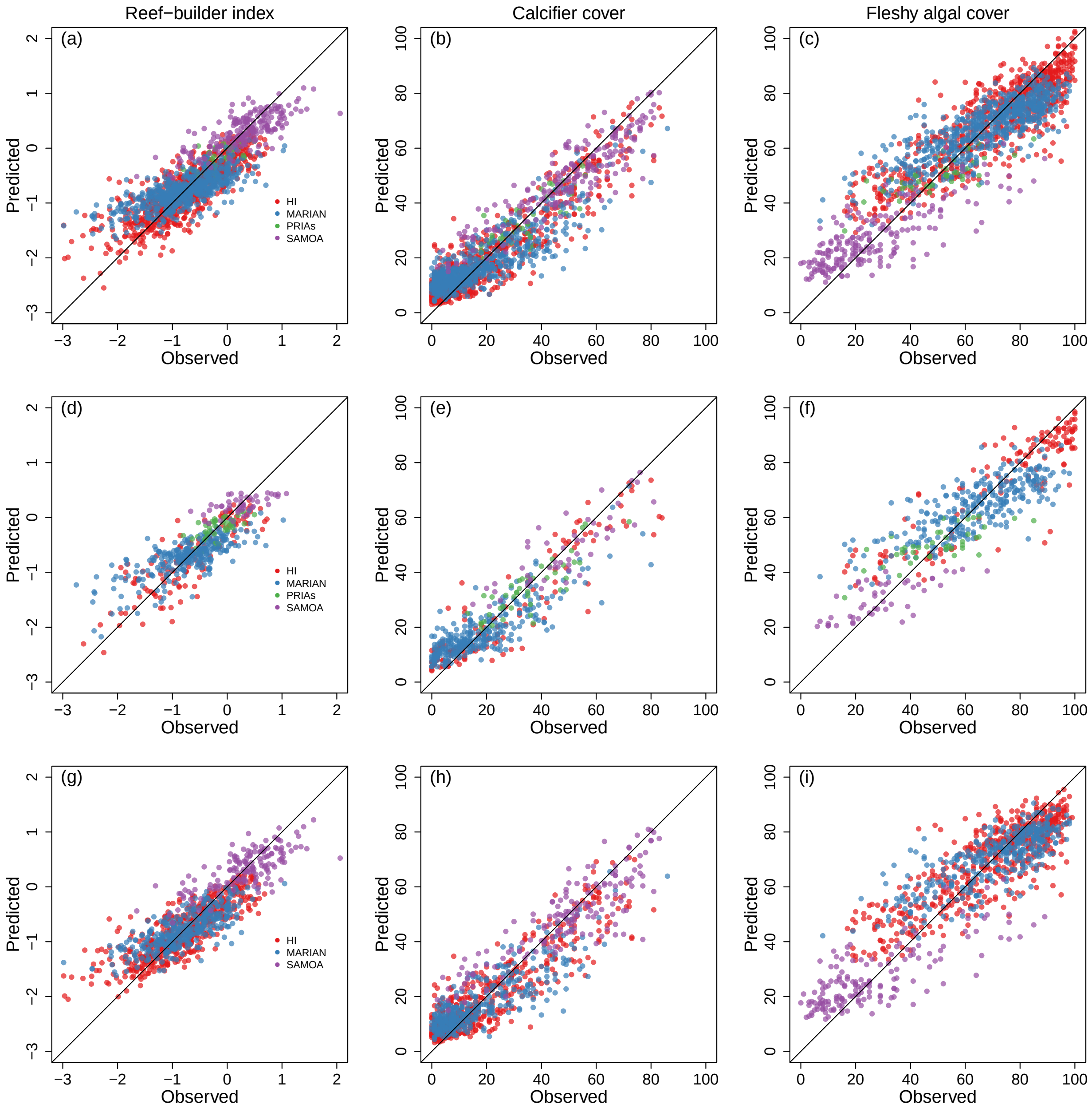

Figure A3. Model fit from BRTs. Observed and predicted values for each benthic response variable (columns: reef-builder index, calcified cover, fleshy algal cover) from each modelled dataset (rows: all sites, inhabited sites, uninhabited sites), with points coloured by their administrative region (red = Marianas, blue = PRIAs, green = Hawaiian, purple = American Samoa) and a 1:1 line.

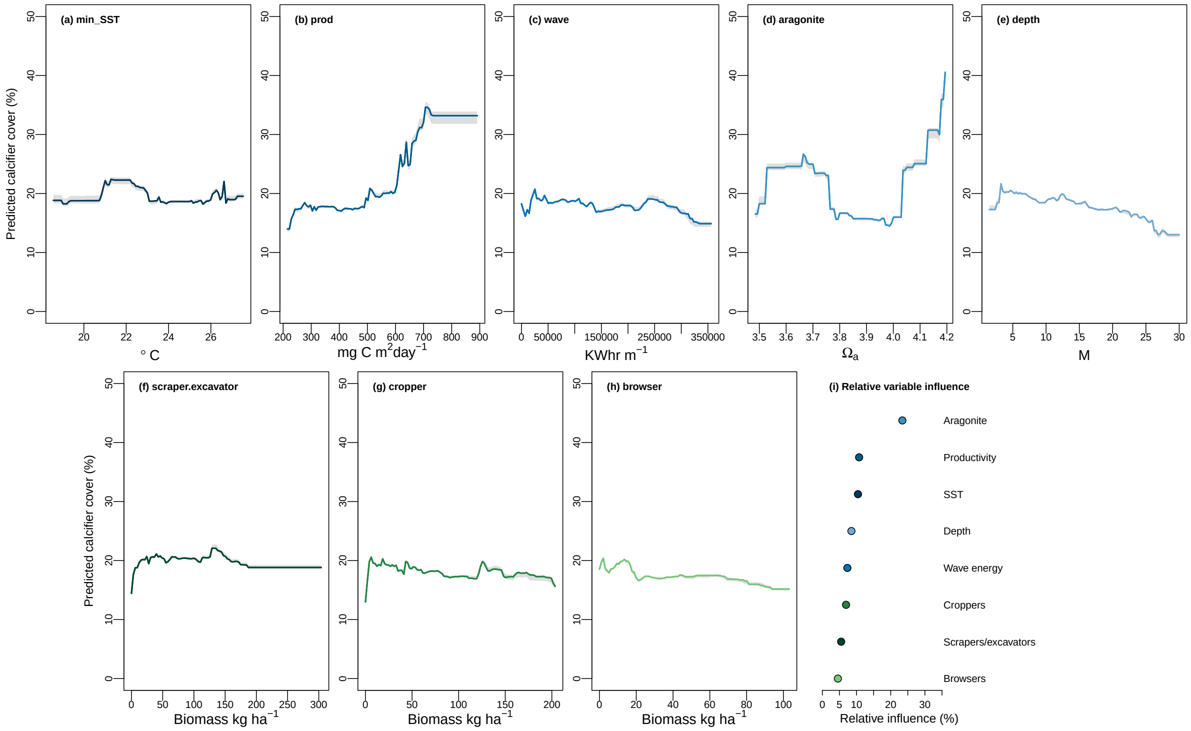

Figure A4. Partial dependence plots and relative importance values for each covariate predicting calcifier cover. Partial dependence plots show predicted change in calcifier cover (%) along the range of each abiotic/bottom-up covariate (a - e) and top-down biotic grazing covariate (f - h). Fitted lines are predicted cover across the range of each selected covariate, holding all other covariates to their mean and with data deciles indicating the distribution of original observations. Grey shaded areas are bootstrapped 95% confidence intervals and red dashed lines are smoothed LOESS functions and shaded areas are 95% uncertainty envelopes generated from bootstrapped model predictions. Relative importance values for each covariate indicated in descending importance (i).

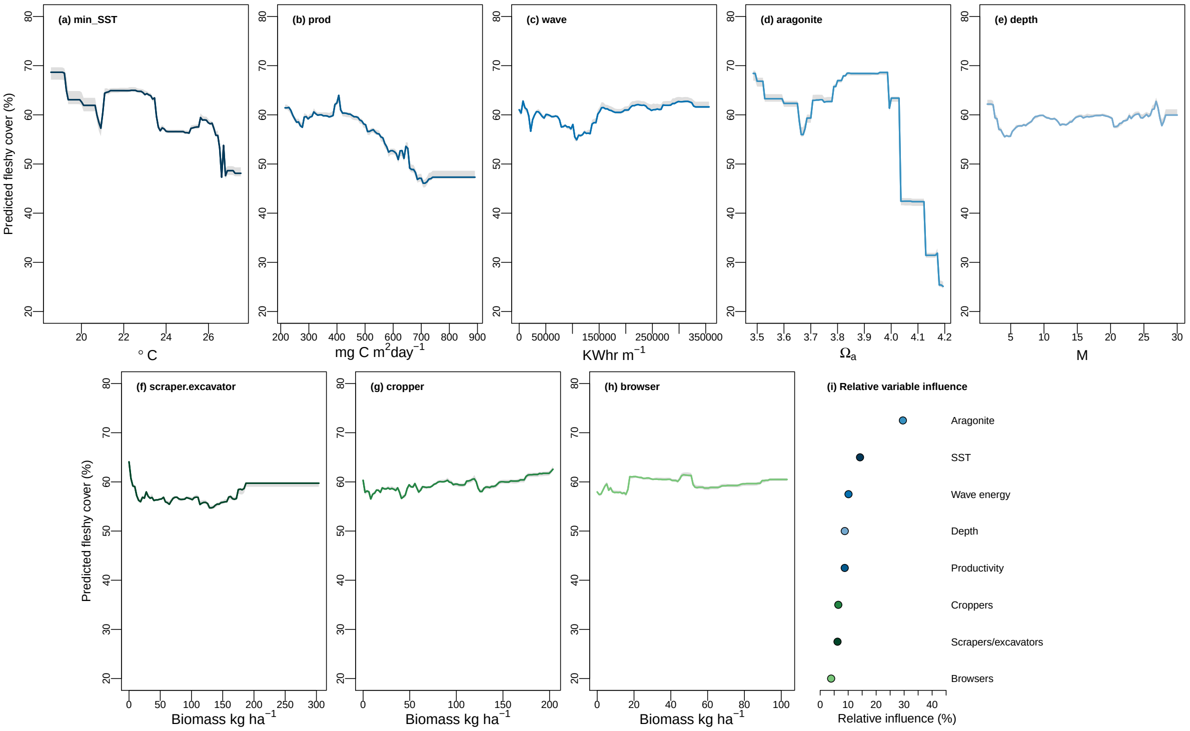

Figure A5. Partial dependence plots and relative importance values for each covariate predicting fleshy algal cover. Partial dependence plots show predicted change in fleshy algal cover (%) along the range of each abiotic/bottom-up covariate (a - e) and top-down biotic grazing covariate (f - h). Fitted lines are predicted cover across the range of each selected covariate, holding all other covariates to their mean and with data deciles indicating the distribution of original observations. Grey shaded areas are bootstrapped 95% confidence intervals and red dashed lines are smoothed LOESS functions and shaded areas are 95% uncertainty envelopes generated from bootstrapped model predictions. Relative importance values for each covariate indicated in descending importance (i).

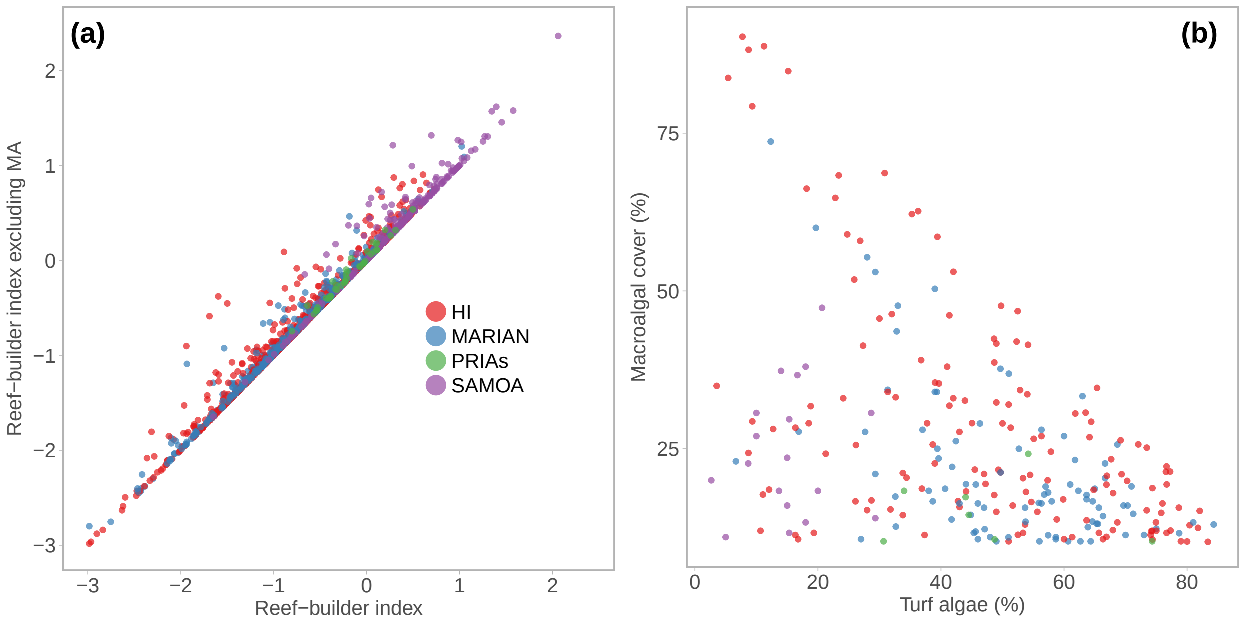

Figure A6. Contribution of macroalgae to reef-builder index and to fleshy algal cover. Reef-builder index estimated without macroalgal cover plotted against observed reef-builder index (a) and relative abundance of macroalgae and turf shown for sites where macroalgae > 10% (258 sites, 16.%% of full dataset) (b). 20 sites in Hawaiian and Marianas archipelagoes have macroalgal cover >50% (1.3% of dataset). Points coloured by their administrative region (red = Marianas, blue = PRIAs, green = Hawaiian, purple = American Samoa).
