# Supplementary material for: Environmental conditions and herbivore biomass determine coral reef benthic community composition: implications for quantitative baselines
Source: Coral Reefs. 2018 Oct 4;37(4):1157–68. doi: 10.1007/s00338-018-01737-w (PMC6404665; doi:10.1007/s00338-018-01737-w)
Supplement: Supplementary file 3 — Supplementary material 3 (DOCX 119 kb) [file 338_2018_1737_MOESM3_ESM.docx]

**Appendix 3 – Supplementary Methods**

*Underwater visual census data*

UVCs and PQs were conducted at unreplicated sites stratified across three depth bins (shallow, 0-6 m; medium, 6-18 m; deep, 18-30 m) on the forereef habitat (also known as reef slope or outer reef) of each atoll/island. Fish communities were surveyed with stationary point counts, in which paired divers surveyed two adjacent 7.5 m radius cylinders centered at 7.5 and 22.5 m on a 30 m transect positioned along a depth contour (2 x 7.5 m radius cylinders = 353.4 m^2^ area surveyed per site). For the first five minutes of each survey, each diver compiled a list of all fish species observed in their cylinder, then sized (total length to nearest cm) and enumerated each recorded species on that list. Following fish surveys, benthic photographs were taken at 1 m intervals along the UVC transect, using a Canon camera attached to a monopod 1 m above the substrate and capturing an area of ~ 0.7 m^2^. Survey procedures are described in further detail in Ayotte et al. (2015) and McCoy et al. (2015). Benthic photographs were analysed by randomly projecting a 5 x 2 grid onto each photo and identifying the benthic organism at each intersecting grid line (10 points) for broad taxonomic groups (hard corals, soft corals, CCA, turf algae, fleshy macroalgae, calcareous macroalgae, calcifying invertebrates). The proportion of each benthic group was converted to a mean percent cover estimates for each site.

*Herbivore biomass processing*

For herbivorous fishes, individual length observations were converted to masses using published length ~ weight relationships (Kulbicki et al. 2005, Froese & Pauly 2016), before generating site-level biomass estimates (kg ha^-1^) by summing fish masses observed by divers in each UVC survey and dividing by the area surveyed. Small juvenile fishes likely only have minimal contributions to grazing, bioerosion and sedimentation processes on coral reefs (Lokrantz et al. 2008) and may also be underestimated in visual surveys (Bozec et al. 2011). Consequently, we excluded fish < 10 cm from our analyses. Infrequent sightings of large herbivore schools that are difficult to count accurately can produce exceptionally high biomass estimates that are not reflective of the grazing biomass at a single site. To prevent these observations from having a disproportionate influence on our predictive models we capped site-level biomass outliers at 95% of the interquartile range, treating each island group separately (Heenan et al. 2016).

*Remote sensing predictor processing*

For productivity estimates, we avoided introducing bias associated with increased reflectance in shallow water areas by excluding cells < 30 m depth from site-level estimates (Gove et al. 2013). We estimated the mean productivity value per site by averaging productivity values across the cell containing the site and the closest 3 neighbouring cells within 9.3 km of either the shoreline or site (if not excluded based on depth) (Yeager et al. 2017).

Site-level wave energy estimates were estimated by creating radial lines in 1° bins unobstructed by land for at least 100 km to give a site's incident wave swath (e.g. Ekebom et al. 2003, Burrows et al. 2008, Chollett & Mumby 2012). We calculated wave power in each degree bin by summing annual wave power values of the closest Wave Watch III pixel with the same direction as the degree bin. Site-level cumulative wave power estimates were calculated by integrating wave power for each degree bin over the entire incident wave swath and averaging annual sums across 1979-2010 (KWhr m^-1^).

*Boosted regression tree performance*

We ensured that boosted regression trees were robust to possible biases introduced by collinearity between predictors and spatial autocorrelation between neighbouring reef sites. Pairwise correlations among predictor covariates indicated moderate collinearity among abiotic covariates (maximum Pearson’s correlation: r = 0.73 for productivity with aragonite saturation state) but no instances of collinearity between abiotic and grazing covariates that might bias our model predictions (Dormann et al. 2007). We also fitted interactions between pairwise predictors, whereby each response variable was predicted across both covariates (holding other covariates at their mean) and then linearly regressed against the same two covariates. Interaction strength was quantified by the mean squared error value from regression residuals, with higher values indicating stronger interactions between predictors. To account for spatial autocorrelation, we produced an autocovariate for each response variable by weighting response values by the geographical distance between neighbouring sites (inverse distance weighting) (*spdep* package) (Bivand et al. 2015), which was subsequently included as an explanatory covariate (Crase et al. 2012). Remaining spatial autocorrelation was assessed by estimating Moran’s *I* coefficient from the model residuals (Diniz-Filho et al. 2003).

**Table A2**. Characteristics of herbivore feeding groups. Each herbivorous fish species observed by CREP was assigned one of four feeding groups, defined following established classifications (References). References: Bellwood et al. (2004); Cheal et al. (2013); Deith (2014); Edwards et al. (2014); Green & Bellwood (2009); Heenan & Williams (2013); Nash et al. (2015).

|  | Feeding group | Definition | Functional role | Families | Species (n) |  |
| --- | --- | --- | --- | --- | --- | --- |
|  | **Cropper** | Graze on turf algae and rarely impact the reef substrate. May also gain feed upon detritus. | Prevent algal overgrowth and clear settlement space to promote coral recruitment | Acanthuridae, Pomacanthidae, Pomacentridae, Monacanthidae, Blenniidae, Siganidae | *Acanthurus achilles, Acanthurus blochii, Acanthurus dussumieri, Acanthurus guttatus, Acanthurus leucocheilus, Acanthurus leucopareius, Acanthurus lineatus, Acanthurus maculiceps, Acanthurus nigricans, Acanthurus nigricauda, Acanthurus nigrofuscus, Acanthurus nigroris, Acanthurus olivaceus, Acanthurus pyroferus, Acanthurus triostegus, Acanthurus xanthopterus, Blenniella chrysospilos, Cantherhines sandwichiensis, Centropyge bicolor, Centropyge bispinosa, Centropyge flavissima, Centropyge heraldi, Centropyge loricula, Centropyge potteri, Centropyge shepardi, Centropyge vrolikii, Cirripectes obscurus, Cirripectes polyzona, Cirripectes vanderbilti, Cirripectes variolosus, Plectroglyphidodon lacrymatus, Plectroglyphidodon sindonis, Siganus argenteus, Siganus punctatus, Siganus spinus, Stegastes albifasciatus, Stegastes aureus, Stegastes fasciolatus, Stegastes nigricans, Zebrasoma flavescens, Zebrasoma rostratum, Zebrasoma scopas, Zebrasoma veliferum (42)* |  |
|  | **Scraper** | Graze on turf algae by lightly scraping the surface of carbonate corals | Prevent algal overgrowth and clear settlement space to promote coral recruitment | Scaridae | *Calotomus carolinus, Calotomus zonarchus, Hipposcarus longiceps, Scarus altipinnis, Scarus dimidiatus, Scarus dubius, Scarus festivus, Scarus forsteni, Scarus frenatus, Scarus fuscocaudalis, Scarus ghobban, Scarus globiceps, Scarus niger, Scarus oviceps, Scarus psittacus, Scarus rubroviolaceus, Scarus schlegeli, Scarus sp, Scarus spinus, Scarus tricolor, Scarus xanthopleura (21)* |  |
|  | **Excavator** | Large-bodied herbivores which graze on turf algae by removing large portions of carbonate corals. | Bioeroders. Promote coral and CCA settlement by removing dead corals. | Scaridae | *Cetoscarus ocellatus, Chlorurus frontalis, Chlorurus japanensis, Chlorurus microrhinos, Chlorurus perspicillatus, Chlorurus sordidus (6)* |  |
|  | **Browser** | Feed on large macroalgal organisms | Prevent overgrowth by large macroalgae | Acanthuridae, Kyphosidae | *Naso lituratus, Naso unicornis, Naso brachycentron, Naso tonganus, Kyphosus cinerascens, Kyphosus vaigiensis, Kyphosus sandwicensis (7)* |  |
|  |  |  |  |  |  |  |

**References**

Ayotte P, McCoy K, Williams ID, Zamzow J. (2015) Coral Reef Ecosystem Division standard operating procedures: data collection for rapid ecological assessment fish surveys.

Bellwood DR, Hughes TP, Folke C, Nyström M. (2004) Confronting the coral reef crisis. Nature 429: 827–833

Bivand R, Piras G. (2015) Comparing implementations of estimation methods for spatial econometrics. J. Stat. Softw. 63

Bozec Y-M, Kulbicki M, Laloë F, Mou-Tham G, Gascuel D. (2011) Factors affecting the detection distances of reef fish: implications for visual counts. Mar. Biol. 158: 969–981

Burrows MT, Harvey R, Robb L (2008) Wave exposure indices from digital coastlines and the prediction of rocky shore community structure. Mar Ecol Prog Ser 353:1–12

Cheal AJ, Emslie M, Aaron MM, Miller I, Sweatman H. (2013) Spatial variation in the functional characteristics of herbivorous fish communities and the resilience of coral reefs. Ecol. Appl. 23: 174–188

Chollett I, Mumby PJ (2012) Predicting the distribution of Montastraea reefs using wave exposure. Coral Reefs 31:493–503

Crase B, Liedloff AC, Wintle BA. (2012) A new method for dealing with residual spatial autocorrelation in species distribution models. Ecography 35: 879–888

Deith, M.D. (2014). Is an ecosystem driven by its species or their traits? Taxonomic and functional diversity in Pacific coral reef fish communities. Honours thesis, Department of Biology, University of Victoria, Victoria, B.C.

Diniz-Filho JAF, Bini LM, Hawkins BA. (2003) Spatial autocorrelation and red herrings in geographical ecology. Glob. Ecol. Biogeogr. 12: 53–64

Dormann CF et al. (2007) Methods to account for spatial autocorrelation in the analysis of species distributional data: a review. Ecography 30: 609–628

Edwards CB et al. (2014) Global assessment of the status of coral reef herbivorous fishes: evidence for fishing effects. Proc. Biol. Sci. 281: 20131835

Ekebom J, Laihonen P, Suominen T. 2003 A GIS-based step-wise procedure for assessing physical exposure in fragmented archipelagos. *Estuar. Coast. Shelf Sci.* **57**, 887–898.

Froese R, Pauly D. (2016) FishBase

Gove JM, Williams GJ, McManus MA, Heron SF, Sandin SA, Vetter OJ, Foley DG (2013) Quantifying climatological ranges and anomalies for Pacific coral reef ecosystems. PLoS One 8:e61974

Green, A. L. and Bellwood, D. R. (2009). Monitoring functional groups of herbivorous reef fishes as indicators of coral reef resilience - A practical guide for coral reef managers in the Asia Pacific region. IUCN working group on Climate Change and Coral Reefs. IUCN, Gland, Switzerland. 70 pages

Heenan A, Williams ID. (2013) Monitoring herbivorous fishes as indicators of coral reef resilience in American Samoa. PLoS One 8: e79604

Heenan A, Hoey AS, Williams GJ, Williams ID. (2016) Natural bounds on herbivorous coral reef fishes. Proc. R. Soc. B 283: 20161716

Kulbicki M, Guillemot N, Amand M. (2005) A general approach to length-weight relationships for New Caledonian lagoon fishes. Cybium 29: 235–252

Lokrantz J, Nyström M, Thyresson M, Johansson C. (2008) The non-linear relationship between body size and function in parrotfishes. Coral Reefs 27: 967–974

McCoy, K., Williams, I., & Heenan, A. (2015). A Comparison of Rapid Visual Assessments and Photo- Quadrat Analyses to Monitor Coral Reef Habitats Data Report.

Nash, K. L., Graham, N. A. J., Jennings, S., Wilson, S. K., Bellwood DR. (2015) Herbivore cross‐scale redundancy supports response diversity and promotes coral reef resilience. J. Appl. Ecol. 53: 646–655

Yeager LA, Marchand P, Gill DA, Baum JK, McPherson JM. (2017) MSEC: Queryable global layers of environmental and anthropogenic variables for marine ecosystem studies. *Ecology* (doi:10.1002/ecy.1884)
