# Supplementary material for: Environmental conditions and herbivore biomass determine coral reef benthic community composition: implications for quantitative baselines
Source: Coral Reefs. 2018 Oct 4;37(4):1157–68. doi: 10.1007/s00338-018-01737-w (PMC6404665; doi:10.1007/s00338-018-01737-w)
Supplement: Supplementary file 6 — Supplementary material 6 (DOCX 107 kb) [file 338_2018_1737_MOESM6_ESM.docx]

***Appendix 6 - Caveats and Limitations***

*Reef-builder index performance*

The reef-builder index was strongly influenced by abiotic and biotic conditions, despite being a coarse measure of the relative abundance of calcifiers to algal organisms. As a univariate metric, the index enabled us to detect shifts in benthic cover at a large latitudinal extent, incorporating temperature and productivity gradients that spanned the tropical Pacific while extending Smith et al.’s (2016) study of anthropogenic impacts on the same reefs. However, we were unable to detect compositional shifts within calcifying or algal taxa that might signify the existence of multiple regime states (Jouffray et al. 2015). For example, reefs may be dominated by cropped turf algae that is intensively grazed by large cropper populations (Heenan et al. 2016), which is habitat conducive for coral settlement (Arnold et al. 2010). Alternatively, macroalgal taxa may dominate, which inhibit coral settlement and are associated with browsing herbivores rather than scrapers and excavators (Helyer & Samhouri 2017). Examination of such compositional shifts (i.e. from cropped to uncropped turf, or turf to macroalgal dominance) across large spatial scales will help to identify the local conditions under which alternate regimes exist.

*Grazing pressure proxy*

We cannot discount the fact that weak grazing influences may partially reflect sampling error due to unreplicated, ‘snapshot’ UVC surveys. Allometric scaling of herbivore foraging distances and home ranges with individual body size indicate that large herbivorous fish species - scrapers and excavators - forage across areas up to ~0.5 km^2^ (Nash et al. 2013, 2015). Given that these species also typically school in large aggregations (Green & Bellwood 2009), such behaviours add uncertainty to biomass estimates when surveys are unreplicated and limited to relatively small areas of the reef (~ 350 m^2^), and thus fail to fully characterise the resident fish community. Although we attempted to control for variability in fine-scale biomass estimates by capping biomass outliers, surveys that are replicated through time will provide a more representative measure of grazing biomass. Analyses of replicated ecological surveys would help resolve uncertainty in the relative influences of abiotic and top-down pressures on benthic state at small scales.

*Resolution of remote sensing covariates*

We also note that our analyses were limited to some extent by the resolution of our remotely-sensed covariates and our measurement of wave energy and aragonite saturation state. First, as with all fine-scale coral reef macroecological analyses, temperature and productivity estimates were extracted at coarser resolutions (i.e. at ~21 km^2^) than UVC sites, and thus neighbouring sites were likely to be assigned identical environmental conditions. Although temperature and productivity can vary seasonally (Liechter et al. 2003, 2006), here, both variables were represented by long-term climatology estimates that characterised the latitudinal temperature gradient and productive upwelling regions of the tropical Pacific. Correspondingly, our covariates varied more over large than fine scales and, as such, this resolution mismatch likely had a minimal influence on our predictive models. Second, although our site-level assessment of wave and aragonite conditions on reef benthos is novel, both biophysical covariates were also coarsely resolved. Aragonite climatology was developed at coarse spatial (1 x 1º) and temporal (1972-2013) resolutions, and thus failed to account for seasonal variation (e.g. Kuchinke et al. 2014) or distinguish between benthic and pelagic carbonate sources. As many calcifier-dominant islands were characterised by island-mean aragonite saturation values (e.g. American Samoa), these limitations may have inflated the predictive power of aragonite, particularly in percent cover models which contained marginal spatial autocorrelation. Similarly, our time-integrated method of estimating long-term mean wave energy was unsuitable for quantifying the frequency and magnitude of extreme wave events, which are major physical influences on reef benthic structure (Gove et al. 2015) These data limitations – resolution and unmeasured components of abiotic forces - may partially explain the limitations in the predictive performance of our BRT models, in which ~50% of variation in the reef-builder index was unexplained and explanatory covariates were unable to account for exceptionally high and low reef-builder index values. Development of site-level wave energy anomaly data (e.g. Gove et al. 2015), temporally-explicit SST and productivity data and *in situ* aragonite saturation data will enable further investigation of the relative roles of average and anomalous wave events, seasonality, and local aragonite conditions, and thus improve our understanding of biophysical processes on reefs across scales.

**Appendix 6 references**

Arnold SN, Steneck RS, Mumby PJ (2010) Running the gauntlet: inhibitory effects of algal turfs on the processes of coral recruitment. Mar Ecol Prog Ser 414:91–105

Gove JM, Williams GJ, McManus MA, Clark SJ, Ehses JS, Wedding LM. (2015) Coral reef benthic regimes exhibit non-linear threshold responses to natural physical drivers. *Mar. Ecol. Prog. Ser.* **522**, 33–48

Green AL, Bellwood DR. (2009) Monitoring functional groups of herbivorous reef fishes as indicators of coral reef resilience - A practical guide for coral reef managers in the Asia Pacific region.

Heenan A, Hoey AS, Williams GJ, Williams ID (2016) Natural bounds on herbivorous coral reef fishes. Proc Biol Sci 283:20161716

Helyer J, Samhouri JF (2017) Fishing and environmental influences on estimates of unfished herbivorous fish biomass across the Hawaiian Archipelago. Mar Ecol Prog Ser 575:1–15

Kuchinke M, Tilbrook B, Lenton A. (2014) Seasonal variability of aragonite saturation state in the Western Pacific. *Mar. Chem.* **161**, 1–13

Leichter JJ, Stewart HL, Miller SL. 2003 Episodic nutrient transport to Florida coral reefs. *Limnol. Oceanogr.* **48**, 1394–1407

Leichter JJ, Helmuth B, Fischer AM. (2006) Variation beneath the surface: Quantifying complex thermal environments on coral reefs in the Caribbean, Bahamas and Florida. *J. Mar. Res.* **64**, 563–588

Nash KL, Graham N, Bellwood DR. (2013) Fish foraging patterns, vulnerability to fishing and implications for the management of ecosystem function across scales. *Ecol. Appl.* **23**, 1632–1644

Nash, K. L., Graham, N. A. J., Jennings, S., Wilson, S. K., Bellwood DR. (2015) Herbivore cross‐scale redundancy supports response diversity and promotes coral reef resilience. *J. Appl. Ecol.* **53**, 646–655

Smith JE, Brainard R, Carter A, Grillo S, Edwards C, Harris J, Lewis L, Obura D, Rohwer F, Sala E, Vroom PS, Sandin S (2016) Re-evaluating the health of coral reef communities: baselines and evidence for human impacts across the central Pacific. Proc Biol Sci 283:20151985
